# Semi-mechanistic Multiple-Analyte Pharmacokinetic Model for an Antibody-Drug-Conjugate in Cynomolgus Monkeys

**DOI:** 10.1007/s11095-014-1585-y

**Published:** 2014-12-03

**Authors:** Dan Lu, Jin Yan Jin, Sandhya Girish, Priya Agarwal, Dongwei Li, Saileta Prabhu, Randall C. Dere, Ola M. Saad, Denise Nazzal, Neelima Koppada, Saroja Ramanujan, Chee M. Ng

**Affiliations:** 1 Department of Clinical Pharmacology, Genentech, Inc, 1 DNA Way, South San Francisco, California 94080 USA; 2 Department of Preclinical and Translational Pharmacokinetics and Pharmacodynamics, Genentech, Inc, South San Francisco, California USA; 3 Department of Bioanalytical Sciences, Genentech, Inc, South San Francisco, California USA; 4 Perelman School of Medicine, University of Pennsylvania, Philadelphia, Pennsylvania USA; 5 Children’s Hospital of Philadelphia, CTRB Building Room 4010, 3501 Civic Center Blvd, Philadelphia, Pennsylvania 19104 USA

**Keywords:** antibody-drug conjugate, integrated modeling, multiple analytes, pharmacokinetics

## Abstract

**Purpose:**

A semi-mechanistic multiple-analyte population pharmacokinetics (PK) model was developed to describe the complex relationship between the different analytes of monomethyl auristatin E (MMAE) containing antibody-drug conjugates (ADCs) and to provide insight regarding the major pathways of conjugate elimination and unconjugated MMAE release *in vivo*.

**Methods:**

For an anti-CD79b-MMAE ADC the PK of total antibody (Tab), conjugate (evaluated as antibody conjugated MMAE or acMMAE), and unconjugated MMAE were quantified in cynomolgus monkeys for single (0.3, 1, or 3 mg/kg), and multiple doses (3 or 5 mg/kg, every-three-weeks for 4 doses). The PK data of MMAE in cynomolgus monkeys, after intravenous administration of MMAE at single doses (0.03 or 0.063 mg/kg), was included in the analysis. A semi-mechanistic model was developed and parameter estimates were obtained by simultaneously fitting the model to all PK data using a hybrid ITS-MCPEM method.

**Results:**

The final model well described the observed Tab, acMMAE and unconjugated MMAE concentration-time profiles. Analysis suggested that conjugate is lost via both proteolytic degradation and deconjugation, while unconjugated MMAE in systemic circulation appears to be mainly released via proteolytic degradation of the conjugate.

**Conclusions:**

Our model improves the understanding of ADC catabolism, which may provide useful insights when designing future ADCs.

**Electronic supplementary material:**

The online version of this article (doi:10.1007/s11095-014-1585-y) contains supplementary material, which is available to authorized users.

## Introduction

Antibody-drug conjugates (ADCs) are a novel class of therapeutic agents that enable targeted delivery of cytotoxic chemotherapeutic agents while reducing their systemic exposure by linking the cytotoxic drug to a targeted monoclonal antibody (mAb). ADCs combine the targeting property and favorable pharmacokinetics (PK) of a mAb with the cytotoxic properties of highly potent cytotoxic agents to provide a class of drugs with an improved therapeutic window. Currently, two ADCs, ado-trastuzumab emtansine (Kadcyla^™^) (1) and brentuximab vedotin (ADCETRIS^™^) (2) have been approved by the U.S. Food and Drug Administration, and at least 20 investigational ADCs are in different stages of development for treating solid tumors and hematological malignancies (3,4). The linker component, which links the antibody with the cytotoxic agent, is an essential part of ADC design and determines the stability of an ADC *in vivo* (3). Several linker types, including acid labile linker, protease labile linker, non-cleavable linker and disulfide linker have been used for these ADCs (3).

**Fig. 1:**
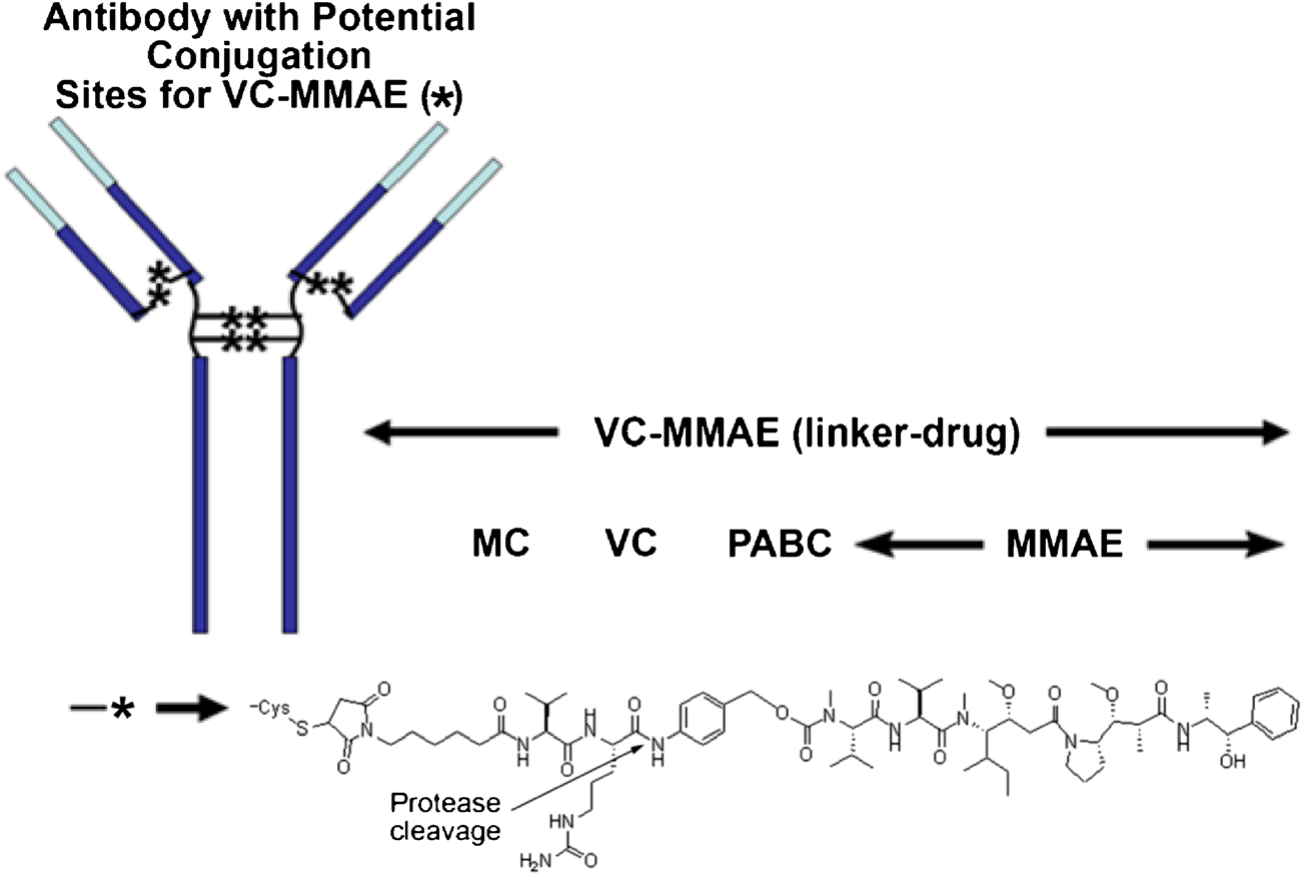
Chemical structure of MMAE-ADCs with MC-VC-PABC linker (5). MC: maleimidocaproyl; MMAE: monomethyl auristatin E; PABC: p-aminobenzoyloxycarbonyl; VC: valine-citrulline

Several monomethyl auristatin E (MMAE) containing ADCs using the protease-labile di-peptide linker (maleimidocaproyl-valine-citrulline-p-aminobenzoyloxycarbonyl [MC-VC-PABC]) (Genentech data on file) are in clinical development (Fig. 1). Each MMAE containing ADC is administered as a mixture of components with different drug to antibody ratios (i.e., different DAR species), ranging from 0 to 8 molecules of cytotoxic drugs per antibody molecule, with an average DAR of approximately 3.5-3.6 (Genentech data on file).

**Fig. 2:**
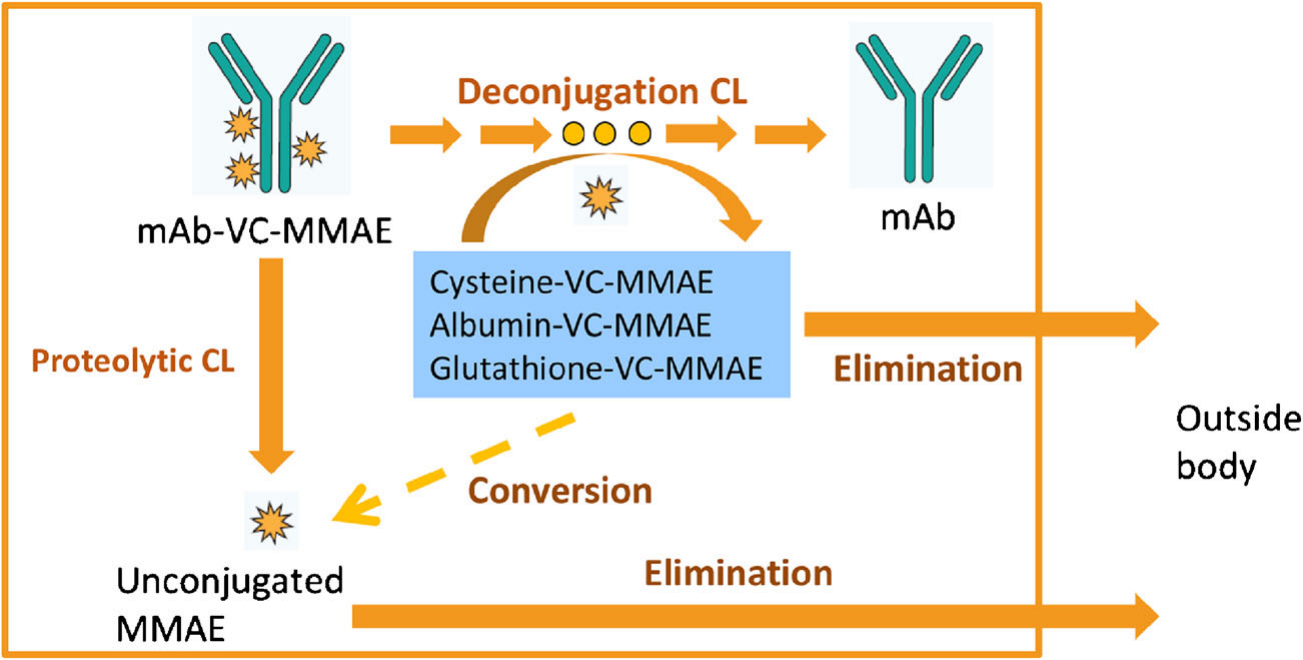
Hypothetical MMAE containing ADC catabolism pathways. CL: clearance; mAb: monoclonal antibody; MMAE: monomethyl auristatin E; VC: valine-citrulline

ADCs demonstrate a unique mechanism of action and complex composition and their distribution, catabolism and elimination processes are not yet well understood. Hypothetically, an ADC may be eliminated via multiple complex pathways that are related to the antibody component (e.g., proteolytic degradation pathway) and the physiochemical properties of the linkers (e.g., deconjugation pathway) (6,7). Similar to typical mAbs, ADCs can undergo proteolytic degradation mediated by target-specific or nonspecific cellular uptake and the neonatal Fc receptor (FcRn)-mediated recycling process, to break down the ADC and generate the unconjugated cytotoxic drug. Furthermore, ADCs may undergo chemical and enzymatic processes (e.g., maleimide exchange) that deconjugate the drug molecules from the antibody component (8), and generate the unconjugated drugs or other related catabolites. This process converts high DAR species to low DAR species or unconjugated antibody. With ADC catabolism, the concentrations of individual DAR species change with time, and the average DAR decreases over time. This was observed for trastuzumab emtansine (T-DM1), an ADC composed of trastuzumab and the cytotoxic drug DM1 via a non-cleavable thioether linker, when administered to cynomolgus monkeys (9). A hypothetical catabolism scheme of a MMAE containing ADC is shown in Fig. 2.

Considering the complex catabolism pathways associated with both the mAb and the drug component post ADC administration, multiple analytes were measured in systemic circulation to assess the pharmacokinetic (PK) properties of an ADC. For the MMAE containing ADCs, these analytes usually include total antibody (Tab) (sum of conjugated, partially unconjugated and fully unconjugated antibody), conjugate (evaluated as antibody-conjugated MMAE, acMMAE) and unconjugated MMAE. Preclinical studies suggest that the toxicity profile of an MMAE containing ADC is consistent with the toxicity profile of MMAE, including reversible bone marrow toxicity and associated hematopoietic changes (Genentech data on file). Both the conjugated MMAE and unconjugated MMAE in the systemic circulation and/or tissue may be associated with antineoplastic efficacy and/or with toxicity. Therefore, it is important to understand the disposition pathway of the conjugate and the release mechanism of unconjugated MMAE into the systemic circulation.

The multiple-analyte integrated PK model was explored for other ADCs such as T-DM1. A semi-mechanistic integrated PK model which assumed sequential deconjugation from high to low DAR species, was developed to describe the PK of T-DM1 conjugate and total trastuzumab after T-DM1 administration in preclinical studies (9–11). This model was then translated to a semi-mechanistic population PK model with multiple transit compartments to characterize T-DM1 and total trastuzumab PK in breast cancer patients (11). A simplified model was developed that used a one-step deconjugation process to convert T-DM1 to unconjugated trastuzumab (10), this successfully described the population pharmacokinetics of T-DM1 and total trastuzumab in cancer patients. These semi-mechanistic integrated models supported the inclusion of both proteolytic degradation and deconjugation as important clearance pathways in the hypothetical scheme of T-DM1 catabolism. However, the PK of the unconjugated cytotoxic drug DM1, an important component of the ADC, was not integrated into these models, primarily because the DM1 assay quantifies all disulfide bound forms of DM1 instead of only unconjugated DM1, and most of the observed unconjugated DM1 concentrations were below the quantitation limit of the assay. As a result, none of these models provided insight into the disposition and major formation route of the unconjugated drug toxin measured in systemic circulation.

CD79b is a signaling component of B-cell receptor restricted to mature B cells, except for plasma cells (12). It is also expressed in nearly all types of B cell hematologic malignancies, including non-Hodgkin’s lymphoma (NHL) and chronic lymphocytic leukemia (CLL) (12). Antibodies bound to CD79b are rapidly internalized, making CD79b ideal for targeted delivery of cytotoxic agents conjugated to anti-CD79b mAbs (12–15). An anti-CD79b-MMAE-containing ADC (12), using a species-specific antibody that binds to human CD79b, is currently in clinical development to treat NHL (5). A surrogate form of this ADC that binds to monkey CD79b receptors was developed for preclinical studies.

The objective of this study was to use the preclinical data of the surrogate anti-CD79b ADC to develop a semi-mechanistic integrated model that can simultaneously describe PK of multiple analytes, including Tab, conjugate, and unconjugated MMAE, for MMAE containing ADCs based on the hypothetical catabolism scheme (Fig. 2). This model was then used to investigate the relationships among the Tab, conjugate, and unconjugated MMAE exposures in order to understand the plausible pathways of ADC catabolism and unconjugated MMAE formation in systemic circulation.

## Materials and Methods

### Antibody Drug Conjugates

The anti-CD79b surrogate ADC, DCDS5017A, has an average DAR of ~3.5 (i.e., average 3.5 MMAE molecules per antibody molecule) (Genentech data on file). The initial distribution of various DAR species in the dosing solution was quantified by hydrophobic interaction chromatography technology (HIC). The molecular weight for MMAE is 718 Daltons and the molecular weight for DCDS5017A is 145239 Daltons (Genentech data on file).

### Pharmacokinetic Data in Cynomolgus Monkeys

The PK of DCDS5017A was evaluated in cynomolgus monkeys after IV administration of a single dose. Three dose levels (0.3, 1 and 3 mg/kg) were evaluated, and 4 monkeys were assessed at each dose level. The PK of three analytes, Tab, acMMAE and unconjugated MMAE were quantified in this PK study. Blood samples were collected pre-dose, at 5 min, and at 4, 12, 24, 72, 168, 336, 504, 672, 840 and 1008 h post-dose. The PK of DCDS5017A was also evaluated in cynomolgus monkeys administered repeated 3 or 5 mg/kg doses every-three-weeks (q3w) for 4 doses. Ten monkeys were assessed at each dose level. The PK of Tab and unconjugated MMAE were quantified pre-dose and at 0.25, 6, 24, 72, 168, 336, and 504 h after the first and the fourth (last) q3w doses, as well as pre-dose and 0.25 h after the second and third q3w doses.

In addition, the PK of unconjugated MMAE was assessed in monkeys administered a single IV injection at doses of 0.03 or 0.063 mg/kg MMAE. Ten monkeys were assessed at each dose level. PK samples were collected pre-dose, at 2, 10, and 30 min post-dose, and at 1, 3, 10, 24, 48, 168, 240 and 504 h post-dose.

The study protocol was approved by the Testing Facility Institutional Animal Care and Use Committee (IACUC) prior to dose administration.

### Bioanalytical Methods

**Fig. 3:**
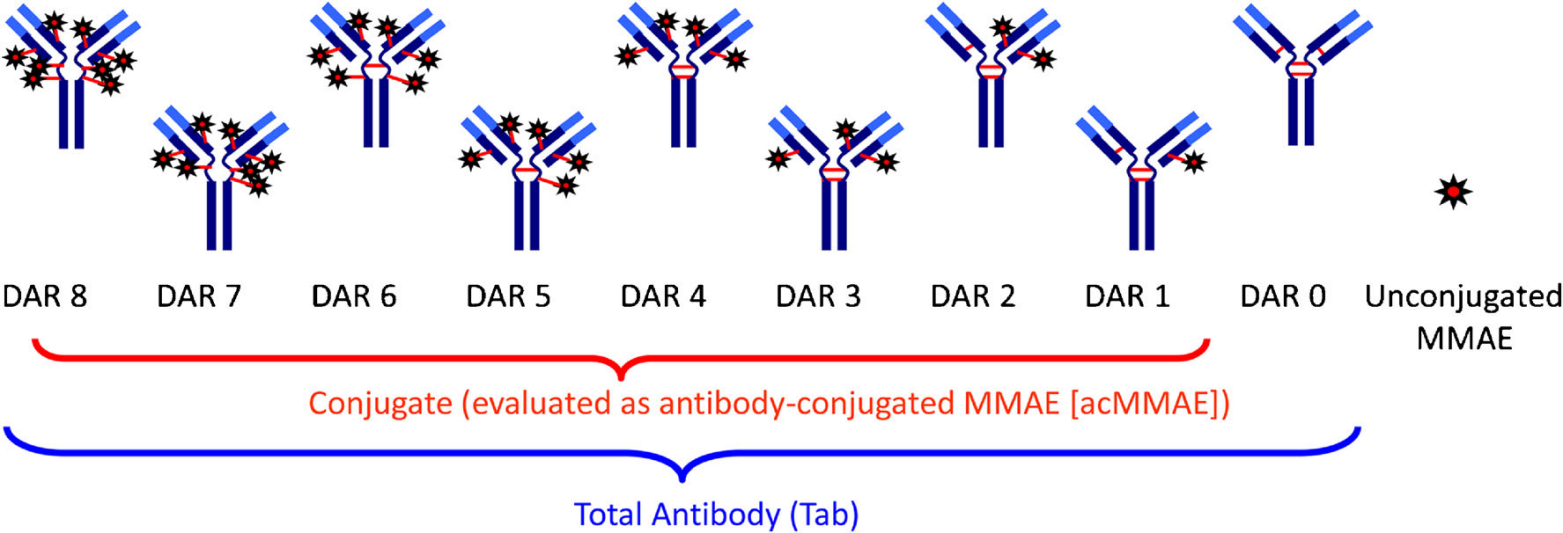
Analytes measured for MMAE containing ADCs. DAR: drug to antibody ratio; MMAE: monomethyl auristatin E

Figure 3 shows the components of the three key PK analytes measured: Tab, conjugate (evaluated as acMMAE) and unconjugated MMAE. The bioanalytical methods for Tab and acMMAE quantify the total concentrations from a heterogeneous mixture of various DAR species (16). Tab concentrations (i.e., the sum of concentrations of all DAR species including fully conjugated, partially deconjugated and fully deconjugated antibody) were measured in serum samples using a validated enzyme linked immunosorbent assay (ELISA) method. The acMMAE concentrations were measured in plasma samples using a method consisting of protein A-affinity capture of the conjugate from plasma followed by enzyme-mediated release of MMAE and quantitative liquid chromatography-tandem mass spectrometry (LC-MS/MS). Unconjugated MMAE was measured in plasma samples using a validated LC-MS/MS method. In cynomolgus monkey studies, the assay lower limits of quantitation (LLOQ) were 0.05 nM (0.0359 ng/mL) for unconjugated MMAE, and 0.195 nM (0.140 ng/mL) for acMMAE, and the minimum quantifiable concentration (MQC) for Tab was 0.06 μg/mL.

### Model Development and Data Analysis

A semi-mechanistic multi-compartment integrated model was developed for simultaneous fitting of Tab, acMMAE and unconjugated MMAE PK data. In this model, the initial dose input for each DAR compartment for the conjugate was based on the initial distribution of various DAR species in the dosing solution, as measured by HIC. The following percentages were obtained for each DAR species: DAR = 0, 5.235%; DAR = 1, 0.61%; DAR = 2, 30.51%; DAR = 4, 48.35%; DAR = 6, 12.755%; DAR = 8, 2.545% (Genentech data on file). The conjugate distribution was described by a two-compartmental model; the conjugate elimination was assumed to be via both the proteolytic degradation and deconjugation pathways. Conjugate was assumed to deconjugate only one MMAE at a time to sequentially convert from DAR_n_ moiety to DAR_n-1_ moiety, where *n* = 1, 2, 3, 4, 5, 6, 7, 8. During model development, various models were tested, including proteolytic degradation clearance by either linear and/or Michaelis-Menten (MM) kinetics, changes in proteolytic degradation clearance with DAR, deconjugation in either the central and/or the peripheral compartments, and various relationships of changes in deconjugation rate constant with DAR. Unconjugated MMAE disposition is described by a two-compartmental linear PK model. Both proteolytic degradation and deconjugation of the ADC provide input of unconjugated MMAE into the central compartment of the MMAE disposition model. The Parallel Iterative-2-Stage and Monte-Carlo Expectation-Maximization (ITS-MCPEM) algorithm implemented in the S-ADAPT II (version 1.57) program, an augmented version of ADAPT II with population analysis capabilities, was used to obtain estimates of the final model parameter value (17,18). Inter-subject variability was assumed to have a log-normal distribution. Intra-subject variability was modeled using a proportional error model. Multiple models that fit the formation of unconjugated MMAE in systemic circulation were explored and the best model was chosen based on a log likelihood ratio test for the nested model (Δ Objective Function Value = 10.3 for df = 1, *p* < 0.001) or Schwarz criterion (19) for the non-nested model. The stringent selection criterion of log likelihood ratio test was used because of the inherent random noise associated with the Monte-Carlo sampling technique employed in the MCPEM algorithm (20). S-PLUS (Version 8.1, Seattle, WA) was used to generate graphical output for model fitting and simulations.

## Results

**Fig. 4:**
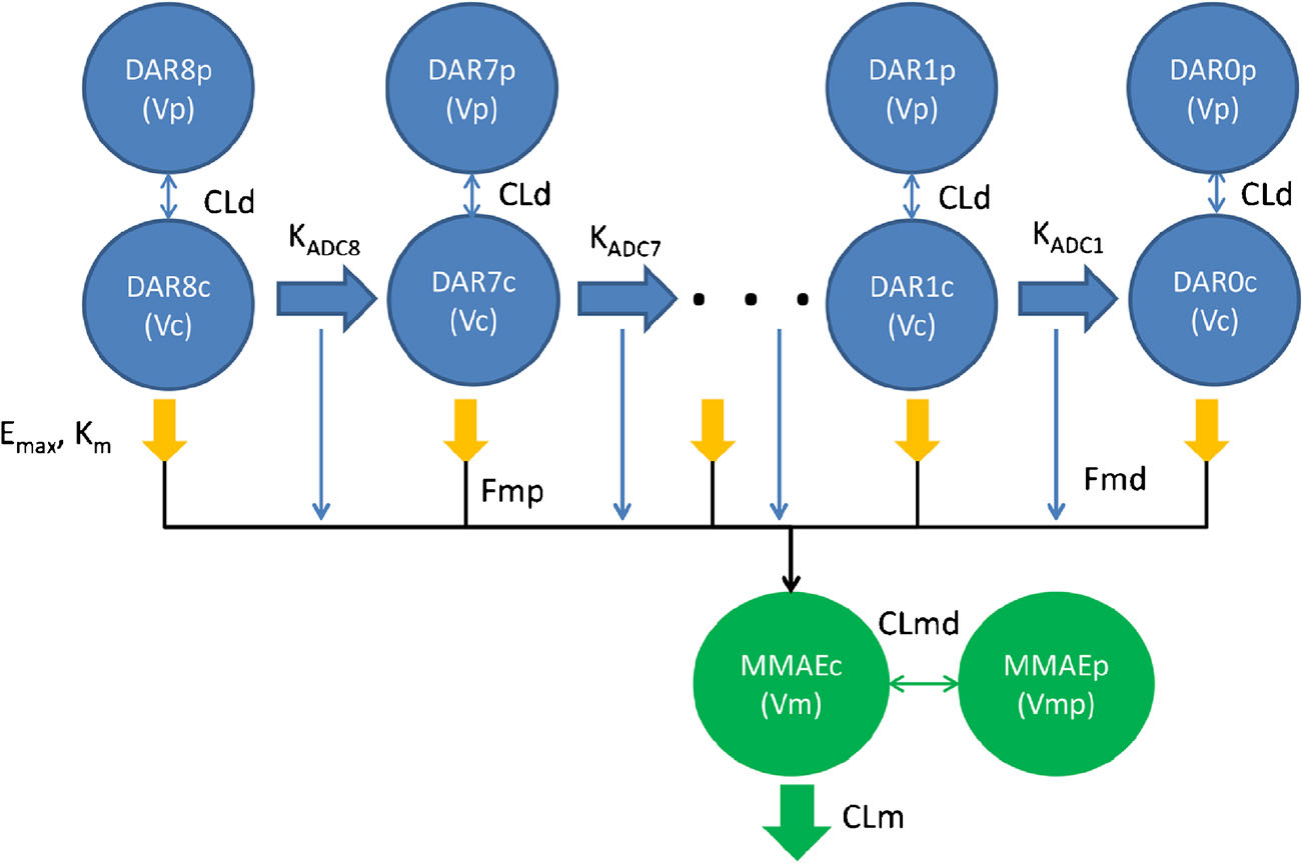
Final model structure of semi-mechanistic multiple-analyte population model for MMAE containing ADCs. ADC: antibody-drug conjugate; CLd: distributional clearance of ADC; CLm: systemic clearance of unconjugated MMAE from the central compartment; CLmd: distributional clearance of unconjugated MMAE; DAR8c, DAR7c, … DAR1c, DAR0c: concentrations of anti-CD79b-MMAE ADC in the central compartment with DAR = 0–8; DAR8p, DAR7p, … DAR1p, DAR0p: concentrations of anti-CD79b ADC in the peripheral compartment with DAR = 0–8; Emax: maximal target-mediated proteolytic degradation rate of ADC; Fmd: fraction of formation of unconjugated MMAE in the central compartment from the deconjugation pathway of ADC; Fmp: fraction of formation to unconjugated MMAE in the central compartment from the proteolytic degradation pathway of ADC; KADC8, KADC7, …KADC1: deconjugation rate constants for each DAR species (DAR = 8, 7, …, 1) of the ADC; Km: concentration of ADC to reach half of the maximal Emax; MMAEc: concentration of unconjugated MMAE in the central compartment; MMAEp: concentration of unconjugated MMAE in the peripheral compartment; Vc: central volume of distribution for ADC; Vp: peripheral volume of distribution for ADC; Vm: central volume of distribution for unconjugated MMAE; Vmp: peripheral volume of distribution for unconjugated MMAE

A total of 447 Tab, 132 acMMAE, and 454 unconjugated MMAE concentration data from 52 monkeys were used for modeling including 32 monkeys receiving DCDS5017A injections and 20 monkeys receiving unconjugated MMAE injections. A mechanism-based multiple-compartment PK model (Fig. 4) was developed that adequately described the PK of Tab, acMMAE and unconjugated MMAE simultaneously. Both proteolytic degradation and deconjugation pathways occur only in the central compartment of the conjugate, as the alternative model with these pathways occurring in the peripheral compartment did not improve the model fitting. The distribution and proteolytic degradation clearances for each DAR moiety were the same, as the alternative model with DAR dependent proteolytic clearance did not improve the model fitting. The proteolytic degradation clearance for the conjugate was described by MM kinetics, as the alternative model with both linear and MM clearance did not improve the model fitting.

The representative equations describing the final semi-mechanistic model are presented below:

1 $$ \frac{{\mathrm{dX}}_{8\mathrm{c}}}{\mathrm{dt}}=-\left(\frac{\mathrm{CLd}}{\mathrm{Vc}}\right)*{\mathrm{X}}_{8\mathrm{c}}+\left(\frac{\mathrm{CLd}}{\mathrm{Vp}}\right)*{\mathrm{X}}_{8\mathrm{p}}-\frac{{\mathrm{Emax}}^{*}{\mathrm{X}}_{8\mathrm{c}}}{{\mathrm{K}\mathrm{m}}^{\ast}\mathrm{V}\mathrm{c}+{\mathrm{X}}_{8\mathrm{c}}}-{8}^{\ast }{{\mathrm{K}}_{\mathrm{ADC}8}}^{*}{\mathrm{X}}_{8\mathrm{c}} $$

2 $$ \frac{{\mathrm{dX}}_{8\mathrm{p}}}{\mathrm{dt}}=\left(\frac{\mathrm{CLd}}{\mathrm{Vc}}\right)*{\mathrm{X}}_{8\mathrm{c}}+\left(\frac{\mathrm{CLd}}{\mathrm{Vp}}\right)*{\mathrm{X}}_{8\mathrm{p}} $$

3 $$ \frac{{\mathrm{dX}}_{7\mathrm{c}}}{\mathrm{dt}}=-\left(\frac{\mathrm{CLd}}{\mathrm{Vc}}\right)*{\mathrm{X}}_{7\mathrm{c}}+\left(\frac{\mathrm{CLd}}{\mathrm{Vp}}\right)*{\mathrm{X}}_{7\mathrm{p}}-\frac{\mathrm{Emax}*{\mathrm{X}}_{7\mathrm{c}}}{\mathrm{K}\mathrm{m}*\mathrm{V}\mathrm{c}+{\mathrm{X}}_{7\mathrm{c}}}+8*{\mathrm{K}}_{\mathrm{ADC}8}*{\mathrm{X}}_{8\mathrm{c}}-7*{\mathrm{K}}_{\mathrm{ADC}7}*{\mathrm{X}}_{7\mathrm{c}} $$

4 $$ \frac{{\mathrm{dX}}_{7\mathrm{p}}}{\mathrm{dt}}=\left(\frac{\mathrm{CLd}}{\mathrm{Vc}}\right)*{\mathrm{X}}_{7\mathrm{c}}-\left(\frac{\mathrm{CLd}}{\mathrm{Vp}}\right)*{\mathrm{X}}_{7\mathrm{p}} $$

5 $$ \frac{{\mathrm{dX}}_{0\mathrm{c}}}{\mathrm{dt}}=-\left(\frac{\mathrm{CLd}}{\mathrm{Vc}}\right)*{\mathrm{X}}_{0\mathrm{c}}+\left(\frac{\mathrm{CLd}}{\mathrm{Vp}}\right)*{\mathrm{X}}_{0\mathrm{p}}-\frac{{\mathrm{E}}_{\max }*{\mathrm{X}}_{0\mathrm{c}}}{\mathrm{K}\mathrm{m}*\mathrm{V}\mathrm{c}+{\mathrm{X}}_{0\mathrm{c}}}+{\mathrm{K}}_{\mathrm{ADC}1}*{\mathrm{X}}_{1\mathrm{c}} $$

6 $$ \frac{{\mathrm{dX}}_{0\mathrm{p}}}{\mathrm{dt}}=\left(\frac{\mathrm{CLd}}{\mathrm{Vc}}\right)*{\mathrm{X}}_{0\mathrm{c}}-\left(\frac{\mathrm{CLd}}{\mathrm{Vp}}\right)*{\mathrm{X}}_{0\mathrm{p}} $$

7 $$ \frac{\mathrm{dXmc}}{\mathrm{dt}}={\mathrm{F}}_{\mathrm{mp}}*{\mathrm{A}}_{\mathrm{mp}}+{\mathrm{F}}_{\mathrm{md}}*{\mathrm{A}}_{\mathrm{md}}-\left(\frac{\mathrm{CLm}}{\mathrm{Vm}}+\frac{\mathrm{CLm}\mathrm{d}}{\mathrm{Vm}}\right)*\mathrm{X}\mathrm{m}\mathrm{c}+\frac{\mathrm{CLm}\mathrm{d}}{\mathrm{Vm}\mathrm{p}}*\mathrm{X}\mathrm{m}\mathrm{p} $$

8 $$ \frac{\mathrm{dXmp}}{\mathrm{dt}}=\frac{\mathrm{CLmd}}{\mathrm{Vm}}*{\mathrm{X}}_{\mathrm{mc}}-\frac{\mathrm{CLmd}}{\mathrm{Vm}\mathrm{p}}*{\mathrm{X}}_{\mathrm{mp}} $$

Where

9 $$ {\mathrm{A}}_{\mathrm{m}\mathrm{p}}=8*\frac{{\mathrm{E}}_{\max }*{\mathrm{X}}_{7\mathrm{c}}}{\mathrm{K}\mathrm{m}*\mathrm{V}\mathrm{c}+{\mathrm{X}}_{8\mathrm{c}}}+7*\frac{\mathrm{E}\mathrm{m}\mathrm{ax}*\mathrm{X}7\mathrm{c}}{\mathrm{K}\mathrm{m}*\mathrm{V}\mathrm{c}+{\mathrm{X}}_{8\mathrm{c}}}+\dots +\frac{{{\mathrm{E}}_{\max}}^{*}{\mathrm{X}}_{0\mathrm{c}}}{{\mathrm{K}}_{\mathrm{m}}*{\mathrm{V}}_{\mathrm{c}}+{\mathrm{X}}_{0\mathrm{c}}} $$

10 $$ {\mathrm{A}}_{\mathrm{md}}=8*{\mathrm{K}}_{\mathrm{A}\mathrm{DC}8}*{{\mathrm{X}}_8}_{\mathrm{c}}+7*{\mathrm{K}}_{\mathrm{A}\mathrm{DC}7}\ *{\mathrm{X}}_{7\mathrm{c}}+\dots +{\mathrm{K}}_{\mathrm{A}\mathrm{DC}1}*{\mathrm{X}}_{1\mathrm{c}} $$

X_nc_ (e.g., X_8c_, X_7c_, etc.,) and X_np_ (e.g., X_8p_, X_7p_, etc.,) represent the amount of the DAR_n_ moiety of ADC in the central and peripheral compartments, respectively. The X_mc_ and X_mp_ represent the amount of unconjugated MMAE in the central and peripheral compartments, respectively. Vc and Vp represent central and peripheral volumes of distribution for the ADC. CLd represents the inter-compartmental clearance. Emax and Km are the Michaelis-Menten kinetic constants for the nonlinear proteolytic degradation of ADC antibody from the central compartment. CLm is the systemic clearance of unconjugated MMAE from the central compartment, and CLmd is the inter-compartmental clearance of unconjugated MMAE. Vm and Vmp are the central and peripheral volumes of distribution for unconjugated MMAE, respectively. Fmd represents the fraction of unconjugated MMAE released from the deconjugation pathway of the ADC in the central compartment, and Fmp is the fraction of unconjugated MMAE released from the proteolytic degradation pathway of the ADC in the central compartment. A_mp_ and A_md_ are the total MMAE formation via ADC antibody proteolytic degradation and deconjugation pathways, respectively. K_ADCn_ (e.g., K_ADC8_, K_ADC7_, etc.) represents the deconjugate rate constant of the DAR_n_ moiety of the ADC. The relationship between deconjugation rate and DAR is best described by the Weibull distribution function presented below:

11 $$ {\mathrm{K}}_{\mathrm{ADCn}}=\mathrm{K}*\frac{\kappa }{\lambda }*{\left(\frac{\mathrm{x}}{\lambda}\right)}^{\left(\kappa -1\right)}*{\mathrm{e}}^{-{\left(\frac{\mathrm{x}}{\lambda}\right)}^{\kappa }} $$

κ and λ are the Weibull constant and *x* = 8-n, where n is the DAR; K is fixed to 1 h^−1^. In this case, the highest conjugation rate was observed with the moiety with the largest number of DAR.

The parameters of the final model are summarized in Table I. All these parameters were estimated with good precision (% coefficient of variation (CV) <50). The Vc for the conjugate (0.0990 L or ~39.6 mL/kg, assuming 2.5 kg as typical body weight of a cynomolgus monkey) was approximately equal to the plasma volume, as expected for a mAb (21,22). The Emax and Km for the nonlinear pharmacokinetics of DCDS5017A were 0.0407 h^−1^ and 0.959 nmol/L, respectively. The λ (scale parameter) and κ (shape parameter) of the Weibull distribution function for the deconjugation rate constant of DCDS5017A were 1.51 and 1.10, respectively. Fmp, the fraction of formation of unconjugated MMAE from the proteolytic degradation pathway of DCDS5017A, was estimated to be 0.783, suggesting that 78.3% of MMAE released from proteolytic degradation contributes to the systemic level of unconjugated MMAE. Fmd, the fraction of formation of unconjugated MMAE from the deconjugation pathway of DCDS5017A was estimated to be 0.0242, suggesting that only 2.42% of the MMAE containing catabolites formed from the deconjugation pathway contributed to the systemic level of unconjugated MMAE. Therefore, the majority of systemic unconjugated MMAE may be released from proteolytic degradation rather than deconjugation of the ADC.

**Table I:** Model Parameters for the Final Integrated Model

Parameters	Population mean (%CV)	Inter-individual variability (%CV)
ADC		
Emax (1/h)	0.0407 (5.5)	0.0945 (25.4)
Km (nmol/L)	0.959 (1.68)	––
Vc (L)	0.0996 (2.6)	0.0118 (36.9)
CLd (L/h)	0.00197 (1.9)	–
Vp (L)	0.183 (8.7)	0.230 (25.4)
λ [Weibull scale parameter]	1.51 (0.9)	–
κ [Weibull shape parameter]	1.10 (0.6)	–
Fmp	0.783 (2.2)	–
Fmd	0.0242 (30.9)	1.78 (34.2)
MMAE		
CLm (L/h)	2.27 (8.3)	0.333 (20.2)
Vm (L)	1.00 (0.3)	–
CLmd (L/h)	23.0 (0.7)	–
Vmp (L)	88.4 (4.8)	0.0670 (31.9)
σ_acMMAE_ [Residual error for acMMAE]	0.257 (4.6)	–
σ_Ab_ [Residual error for Tab]	0.194 (4.1)	–
σ_MMAE_ [Residual error for unconjugated MMAE]	0.327 (8.9)	–

ADC: antibody-drug conjugate; CLd: distributional clearance of ADC; CLm: systemic clearance of unconjugated MMAE from the central compartment; CLmd: distributional clearance of unconjugated MMAE; CV: coefficient of variation; Emax: maximal target-mediated proteolytic degradation rate of ADC; Fmd: fraction of formation of unconjugated MMAE in the central compartment from the deconjugation pathway of ADC; Fmp: fraction of formation to unconjugated MMAE in the central compartment from the proteolytic degradation pathway of ADC; Km: concentration of ADC to reach half of the maximal Emax; MMAE: monomethyl auristatin E; Vc: central volume of distribution for ADC; Vp: peripheral volume of distribution for ADC; Vm: central volume of distribution for unconjugated MMAE; Vmp: peripheral volume of distribution for unconjugated MMAE

For unconjugated MMAE, the elimination and distribution clearance (CLm and CLmd) values were 2.27 L/h and 23 L/h, respectively. The central distribution volume (Vm) was 1.00 L, 10-fold larger than the central distribution volume of DCDS5017A. Unconjugated MMAE distributed widely into tissues with a peripheral tissue volume (Vmp) of 88.4 L. The typical distribution and elimination half-lives of the unconjugated MMAE were estimated to be 0.0272 h and 29.9 h, respectively.

**Fig. 5:**
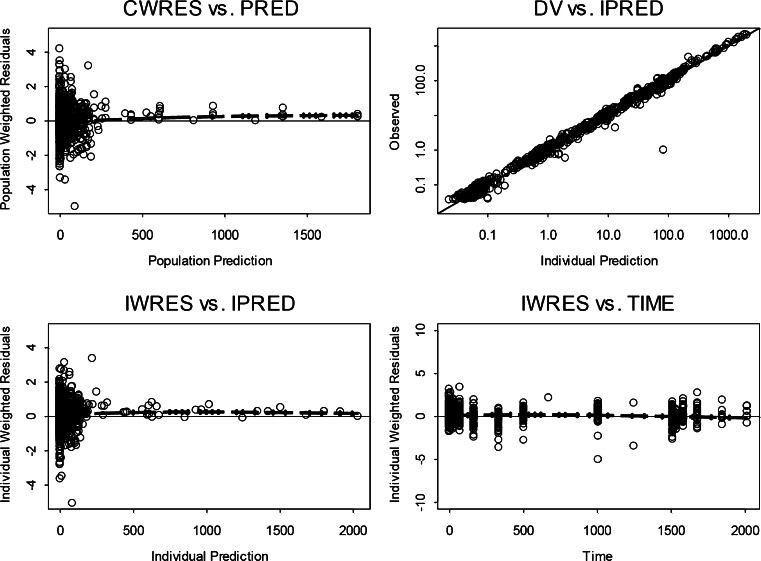
Standard model diagnostic plots for all three analytes. CWRES: conditional weighted residual; DV: dependent variable (refers to PK concentrations); IPRED: individual predictions; IWRES: individual weighted residuals; PRED: population predictions; Time: PK time after the first dose.

**Fig. 6:**
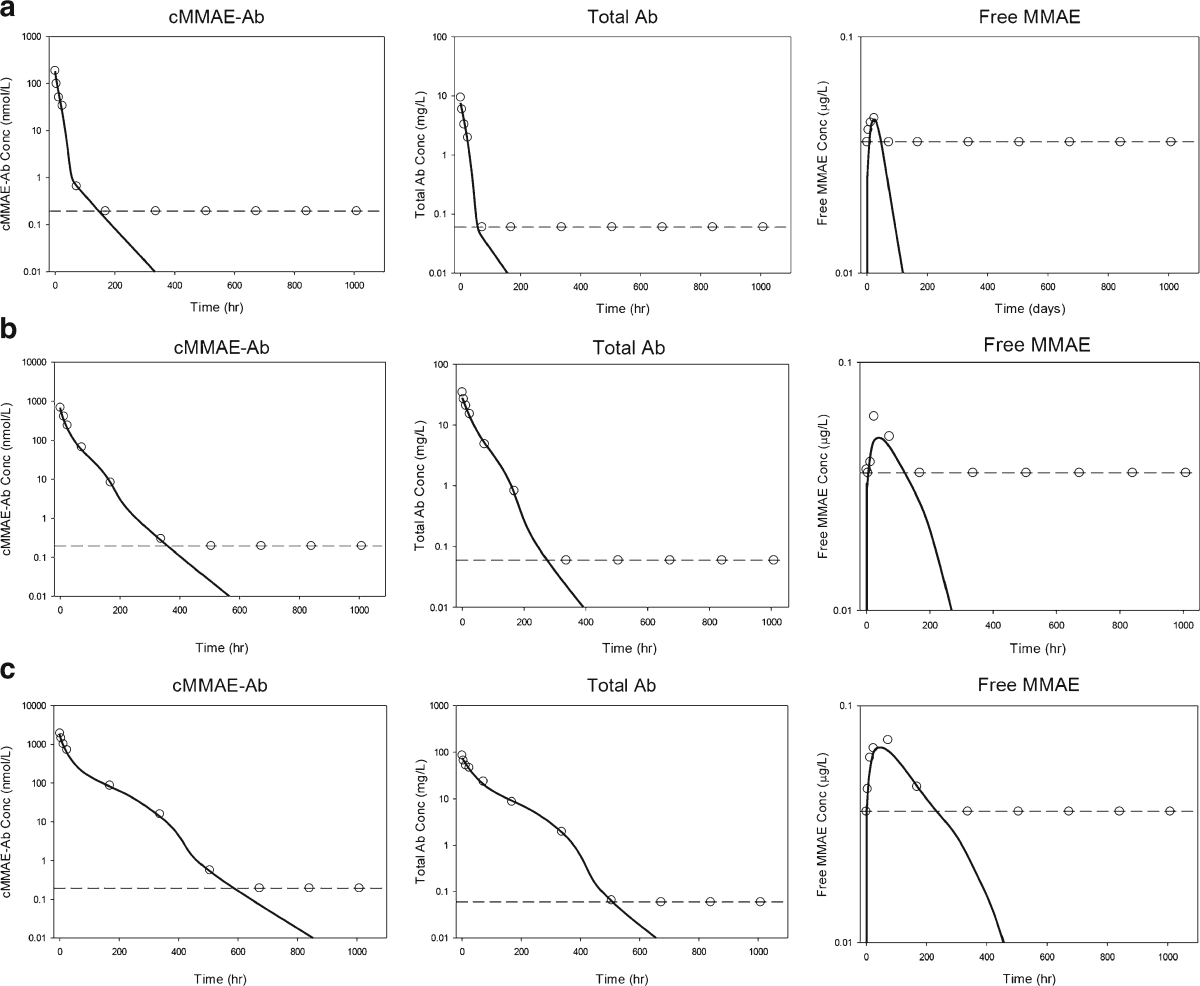
Individual fitting plots for a representative subject after a single dose of (**a**) 0.3 mg/kg, (**b**) 1 mg/kg, and c) 3 mg/kg of anti-CD79b ADC. *Dashed line*: concentration level of ≤ LLOQ or ≤ MQC; *open circle*: observed PK concentrations; *open circles on the dashed line*: observed PK concentrations with ≤ LLOQ or ≤ MQC values; *solid line*: individual model prediction; Tab: total antibody; acMMAE: antibody-conjugated MMAE; MMAE: monomethyl auristatin E.

**Fig. 7:**
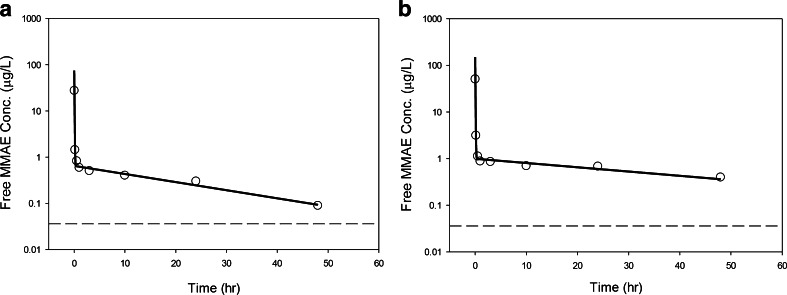
Individual fitting plots for a representative subject after a single dose of (**a**) 0.03 mg/kg, and (**b**) 0.063 mg/kg of IV MMAE. *Dashed line*: concentration level of below quantification limit (BQL); *open circle*: observed PK concentrations; *solid line*: individual model prediction; MMAE: monomethylauristatin E.

The final model reasonably predicted the observed Tab, acMMAE, and unconjugated MMAE PK profiles after administration of either DCDS5017A or unconjugated MMAE. Figure 5 presents individual predicted *versus* observed data for all three analytes in all animals. Generally, there was good agreement between the predicted and observed data, and the diagnostic plots (Fig. 5) of the final model identified no systematic bias. Representative plots of observed and model predicted Tab, acMMAE and unconjugated MMAE concentrations in 3 animals received 0.3, 1, or 3 mg/kg doses of DCDS5017A as shown in Fig. 6. In addition, the model adequately described the observed unconjugated MMAE-time profiles after IV administration of different doses of MMAE (Fig. 7). These plots together with the visual predictive check plots (Supplementary Figure 1) further demonstrate that the final model reasonably described the data.

**Fig. 8:**
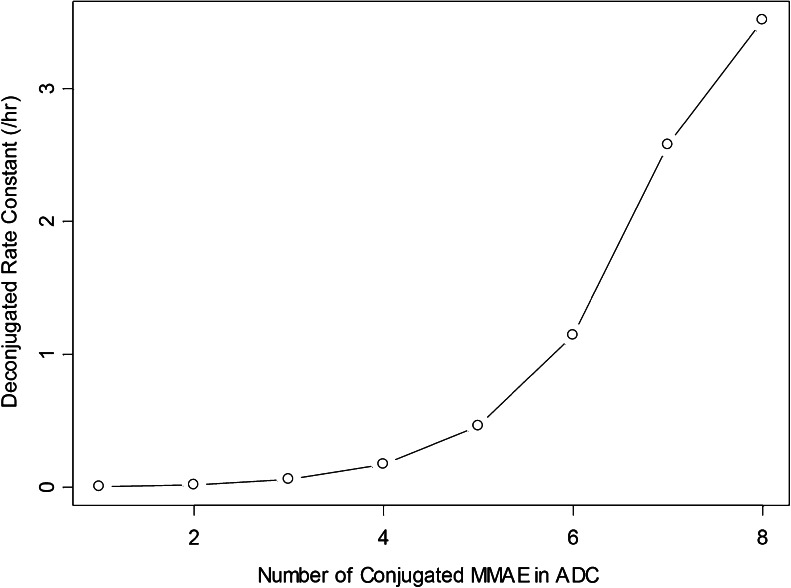
Simulated deconjugation rate based on Weibull model. ADC: antibody-drug conjugate; MMAE: monomethyl auristatin E.

**Fig. 9:**
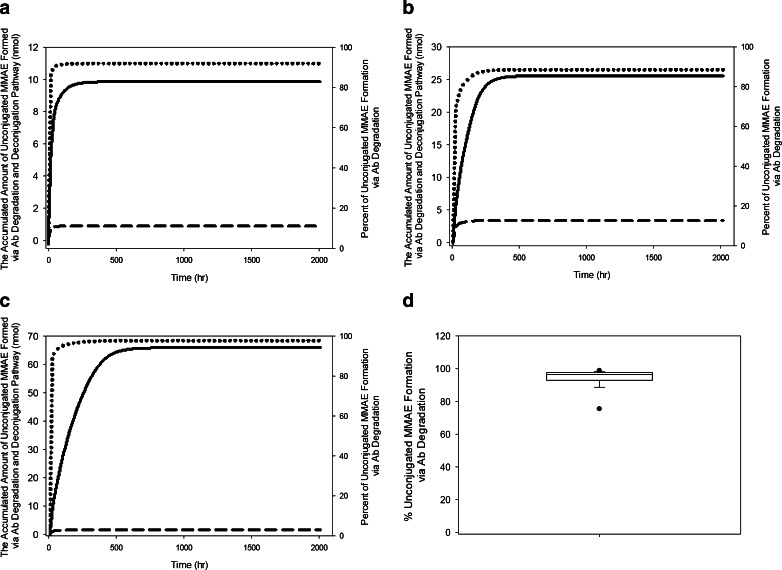
Unconjugated MMAE release-time profile for a representative subject after a single dose of a) 0.3 mg/kg, b) 1 mg/kg, and c) 3 mg/kg of anti-CD79b-MMAE ADC. d) Mean percentage of unconjugated MMAE release due to conjugate proteolytic degradation after a single dose of ADC. *Solid line* – unconjugated MMAE release via Antibody (Ab) proteolytic degradation; *dashed line* – unconjugated MMAE release via deconjugation pathway; *dotted line* – percent of unconjugated MMAE release via conjugate proteolytic degradation pathway.

The Weibull model described best the relationship between deconjugation rate constant and DAR. The deconjugation rate constant decreased from 3.52 to less than 0.01 h^−1^ for DAR_8_ and DAR_1_ species, respectively (Fig. 8), as computed by model parameters. Using the individual parameters obtained from the final model, the amount of unconjugated MMAE formed by both pathways were simulated and calculated. Figure 9a-c show the unconjugated MMAE release-time profiles via deconjugation or the proteolytic degradation pathway from the corresponding animals presented in Fig. 6 that received a single-dose of conjugate in 0.3, 1, and 3 mg/kg. The results suggested that plasma exposure to unconjugated MMAE appears to be affected mainly by the proteolytic degradation pathway rather than the deconjugation pathway. Figure 9d summarizes the percentage of unconjugated MMAE release via conjugate proteolytic degradation in the studied population to be 98% after a single dose of ADC (mean: 94%; range: 61–99%).

## Discussion

After administration to cynomolgus monkeys, MMAE-containing ADCs exhibited complex PK involving multiple analytes including Tab, conjugate (evaluated as acMMAE) and unconjugated MMAE. The model developed in this study was established based on the hypothetical ADC catabolism schemes (Fig. 2). The model well described the observed PK data of MMAE-containing ADC and suggested that the conjugate was eliminated by both deconjugation and proteolytic degradation pathways while the unconjugated MMAE in the systemic circulation was mainly formed by proteolytic degradation of the conjugate.

The model allows a simultaneous estimation of both the deconjugation and proteolytic degradation clearance for the conjugate, because the PK data in cynomolgus monkeys for both Tab and conjugate (acMMAE) are used. Hypothetically, conjugate (acMMAE) is cleared by both deconjugation and proteolytic degradation while total antibody is only cleared by proteolytic degradation. This additional clearance pathway of deconjugation for acMMAE is supported by the observed faster clearance of acMMAE compared to Tab.

To derive the final model in this study, many alternative models were tested during model development. It was reported that the high DAR species are less stable *in vitro*, more prone to aggregation and fragment formation (23) and cleared faster *in vivo* than low DAR species (24,25). Using affinity capture LC-MS, it was found that after T-DM1 administration to cynomolgus monkeys, high DAR species cleared much faster than low DAR species. There was also an increase of DAR1 and DAR0 species after dosing, suggesting the conversion of high DAR species to low DAR species potentially due to deconjugation (25). However, it is not clear whether the data suggest a faster proteolytic degradation, or deconjugation, or both processes, for the high DAR species. By comparing the PK of cAC10-vc-MMAE with DAR of 8, 4, or 2, or unconjugated antibody in SCID mice, using the ELISA assay to quantify the Tab, the clearance of DAR = 8 ADCs was found to be 3–4 fold higher than the DAR = 2 or 4 ADCs and unconjugated antibody, suggesting a potentially faster proteolytic degradation for DAR = 8 ADCs in mice (24). Based on these findings, simpler models using DAR dependent change of proteolytic degradation or deconjugation were constructed first and served as the basis for developing more complex models. In this model, the conjugate distribution was described by a two-compartmental model and eliminated via nonlinear proteolytic degradation clearance, and deconjugated via DAR-dependent simple linear process, and unconjugated MMAE disposition was described by a two-compartmental linear PK model. For the proteolytic degradation clearance, it is possible that instability of the high DAR species may be due to the high proteolytic clearance. However, including the DAR-dependent proteolytic degradation process and additional PK data from the naked CD79b antibody failed to improve the model fit. It is worth noting that in the current study, a mixture of various DAR species were injected into monkeys, and only 15% of the total dose comprised of DAR = 8 or 6 species while the majority were DAR 4 or 2 species (>80% of total dose). It was reported that DAR = 4 or 2 species have relatively similar proteolytic degradation clearance (24) and *in vitro* thermal stability (23), compared to DAR = 8 or 6 species. Given this, and without PK characterization of individual DAR species, the model may not have sufficient data to estimate DAR dependent proteolytic degradation clearance. Therefore, a DAR-independent elimination process similar to several previously published mechanism-based ADC models, was used to describe the proteolytic degradation clearance process of all DAR species in the MMAE-containing ADC (9,11).

For the deconjugation clearance, replacing the constant rate model with the more flexible Weibull model to describe the relationship between deconjugation rate constant and DAR significantly improved the model fit based on Schwarz criterion (Fig. 8). This suggests that conjugate species with high DARs deconjugate much faster than species with low DARs. The MMAE containing ADC with maleimide linkers is manufactured by conjugation through reduced interchain disulfide bonds. This process results in a heterogeneous mixture of ADC molecules with a range of different DARs from 0 to 8 (16). Solvent accessibility and local charge can impact the *in vivo* stability of the conjugate owing to deconjugation by maleimide exchange with reactive thiols in albumin, free cysteine, or glutathione (8). The faster deconjugation rate of high DAR species may be due to a higher probability of unloading toxin molecules when there are relatively high numbers of toxin molecules per molecule of antibody with greater inherent instability impacted by the chemical and structural dynamics of the conjugation site.

Based on the ADC mechanism of action, the proteolytic degradation and deconjugation of the conjugate may occur at tissue sites after cellular uptake to form unconjugated MMAE in the tissue, which back diffuse to the systemic circulation. However, alternative models that incorporated proteolytic or deconjugation clearances of the conjugate in the peripheral compartments (i.e., tissue sites) did not improve the fitting, possibly due to that only serum/plasma concentrations of each analyte were collected in the study. Additional work to understand the proteolytic degradation and deconjugation of ADCs in the tissue would be helpful to assess alternative models.

Similar to mAbs, proteolytic degradation of an ADC is mediated by target-mediated clearance and FcRn mediated recycling after non-specific cellular uptake. For the MMAE containing ADC with MC-VC-PABC linkers, proteolytic degradation may also be impacted by the inherent instability of the conjugate due to the potential disruption of interchain disulfide bonds. In the current model, both linear and nonlinear clearance were tested to describe the proteolytic degradation of the conjugate; however, only a single nonlinear clearance appeared sufficient to describe the data and was used in the final model.

During the development of the final model, the visual predictive check revealed that the presence of several Tab, acMMAE, and MMAE concentrations below LLOQ hampered the ability to characterize the time-course of these analytes using the standard modeling and data handling approach (Fig. 6). Therefore, a likelihood based approach was used to develop the final model, which included all observations with LLOQ values as censored data in the analysis (26). This method has been successfully used to develop several mechanism-based models for describing the anti-therapeutic antibody- and/or receptor-mediated nonlinear PK of a therapeutic monoclonal IgG antibody using PK data with many observations below LLOQ, and allowed us to obtain reliable model parameter estimates by fitting the developed model simultaneously to all data with many MQC observations (20,27).

Both deconjugation and proteolytic degradation of the conjugate were assumed to contribute to the formation of unconjugated MMAE in the systemic circulation, and the fraction of formation from either deconjugation or proteolytic degradation was estimated. With the model parameters of unconjugated MMAE uniquely identified by the PK data in cynomolgus monkeys after IV unconjugated MMAE administration, the input function or formation of systemic unconjugated MMAE after conjugate dosing can be determined. The values for the fraction parameters suggest the proteolytic degradation of the conjugate provides the major source of unconjugated MMAE in the systemic circulation. This could be explained by the fact that deconjugation mainly forms MMAE containing catabolites (e.g., cys-vc-MMAE) which are not measured by the LC-MS/MS method that was developed for quantifying MMAE only. This finding is consistent with results reported by Shah *et al*., using another mechanism-based PK model to predict conjugate and unconjugated drug concentrations in plasma and tumor tissue (28). It was found that clearance of the entire ADC (similar to the proteolytic degradation pathway described herein) seemed to be a higher contributor of released payload (cytotoxic drugs) in plasma than the payload dissociated from intact ADC (similar to the deconjugation pathway described herein). Our current analysis suggests that a decrease of proteolytic degradation clearance may potentially decrease the systemic level of unconjugated MMAE. Proteolytic degradation is composed of target-mediated clearance and FcRn recycling pathway-mediated non-specific cellular uptake and degradation. Target-mediated proteolytic degradation is desirable for efficacy, while non-specific uptake and degradation may be related to toxicity. This analysis suggests that enhancing FcRn-mediated recycling by antibody engineering techniques may be beneficial in reducing non-specific degradation of the conjugate and generation of unconjugated MMAE in systemic circulation, hence decreasing the undesirable toxicity.

One potential limitation of our final model was that the unconjugated MMAE concentrations after ADC dosing were relatively low compared to those observed after single IV MMAE study. Therefore, the potential nonlinearity of the MMAE PK profiles at low concentrations may affect the results in this study. To our knowledge, the PK of the low dose MMAE in cynomolgus monkeys and other species was never reported in the literature besides this paper. However, a recent published PBPK model which assumed linear MMAE PK well described the observed unconjugated MMAE-time profiles after ADC administrations (29). Therefore, we believe that linear MMAE PK assumption used in this study was reasonable in the absence of PK data for low dose MMAE, but more study with low dose MMAE PK data is desirable in order to confirm this study result.

The current model may be further extended to clinical data to understand the quantitative relationship of Tab, acMMAE and unconjugated MMAE in patients. Furthermore, the pharmacokinetic-pharmacodynamic model will also be useful for correlating PK with efficacy and safety endpoints to investigate the systemic exposure of which analyte(s) is best correlated with clinical responses. For brentuximab vedotin, the concentrations of the conjugate in the systemic circulation correlated with the probability of tumor response, neutropenia, and peripheral neuropathy, while the concentrations of unconjugated MMAE did not correlate with efficacy and safety outcomes (30). Additional development of the model to project the tissue concentrations of the conjugate and unconjugated MMAE would be highly valuable to correlate with efficacy and safety profiles in patients.

As the unconjugated antibody species may form after ADC dosing, which may hypothetically exhibit competitive binding to the targets with the drug containing conjugate, it is of interest to estimate the DAR = 0 species PK after ADC dosing. The model applies to simulating the PK profiles of each DAR moiety, specifically the DAR = 0 species PK for various dosing regimens of the conjugate, to assess whether there is accumulation of this species after repeated dosing with different dosing regimens. The final model well described the observed data. However, precise quantification of ADC distribution, catabolism and elimination is still in its infancy and future experiments are needed to confirm the findings of this model analysis. While the final model in this study can describe the complex PK of MMAE-containing ADCs, it is also considered a plausible model based on the current hypothesis and available assay/data for multiple analytes. This model represented an important first step to understand the complex catabolism and unconjugated drug formation for MMAE-containing ADCs and could serve as the base model to build future generations of PK and systems pharmacology models based on our increasing understanding of ADC catabolism.

## Conclusions

Two mechanisms were proposed to describe ADC conjugate catabolism: deconjugation and proteolytic degradation. The integrated semi-mechanistic model reported here adequately described the observed concentration-time profiles of Tab, acMMAE and unconjugated MMAE in cynomolgous monkeys administered anti-CD79b-MMAE containing ADC. Modeling results suggested that conjugated MMAE in ADC is eliminated via both deconjugation and proteolytic degradation; whereas unconjugated MMAE in systemic circulation appears to be released mainly via the proteolytic degradation pathway. This analysis supports the current understanding of ADC catabolism and sheds light on the potential release mechanisms of unconjugated MMAE into the systemic circulation, which may provide valuable insights for future ADC design.

## Electronic supplementary material

Below is the link to the electronic supplementary material.

ESM 1 (GIF 39 kb)

High resolution image (TIFF 707 kb)

## Abbreviations

acMMAE: Antibody conjugated monomethyl auristatin E
ADC: Antibody-drug conjugate
Amd: MMAE formation via ADC antibody deconjugation pathway
Amp: Total MMAE formation via ADC antibody proteolytic degradation pathway
CLd: Distributional clearance of ADC
CLL: Chronic lymphocytic leukemia
CLm: Systemic clearance of unconjugated MMAE from the central compartment
CLmd: Distributional clearance of unconjugated MMAE
CV: Coefficient of variation
DAR: Drug to antibody ratio
ECD: Extracellular domain
ELISA: Enzyme linked immunosorbent assay
Emax: Maximal target-mediated proteolytic degradation rate of ADC
FcRn: Neonatal Fc receptor
Fmd: Fraction of formation of unconjugated MMAE in the central compartment from the deconjugation pathway of ADC
Fmp: Fraction of formation to unconjugated MMAE in the central compartment from the proteolytic degradation pathway of ADC
HIC: Hydrophobic interaction chromatography
ITS-MCPEM: Iterative-2-Stage and Monte-Carlo Expectation-Maximization
K_ADCn_: The deconjugate rate constant of the DAR_n_ moiety of the ADC
Km: Concentration of ADC to reach half of the maximal Emax
LC-MS/MS: Liquid chromatography-tandem mass spectrometry
LLOQ: Lower limit of quantitation
mAb: Monoclonal antibody
MC-VC-PABC: Maleimidocaproyl-valine-citrulline-para-aminobenzoyloxycarbonyl
MM: Michaelis-Menten
MMAE: Monomethyl auristatin E
MQC: Minimum quantifiable concentration
NHL: Non-Hodgkin’s lymphoma
PK: Pharmacokinetic(s)
q3w: Once-every-3-weeks
SE: Standard error
Tab: Total antibody
T-DM1: Trastuzumab emtansine
Vc: Central volume of distribution for ADC
Vm: Central volume of distribution for unconjugated MMAE
Vmp: Peripheral volume of distribution for unconjugated MMAE
Vp: Peripheral volume of distribution for ADC
Xmc: Amount of unconjugated MMAE in the central compartment
Xmp: Amount of unconjugated MMAE in the peripheral compartment
Xnc: amount of the DAR_n_ moiety of ADC in the central compartment
Xnp: Amount of the DAR_n_ moiety of ADC in the peripheral compartment
κ: Weibull shape parameter.
λ: Weibull scale parameter
