# Supplementary figures and images for: Semi-mechanistic Multiple-Analyte Pharmacokinetic Model for an Antibody-Drug-Conjugate in Cynomolgus Monkeys
Source: Pharm Res. 2014 Dec 3;32(6):1907–19. doi: 10.1007/s11095-014-1585-y (PMC4422865; doi:10.1007/s11095-014-1585-y)

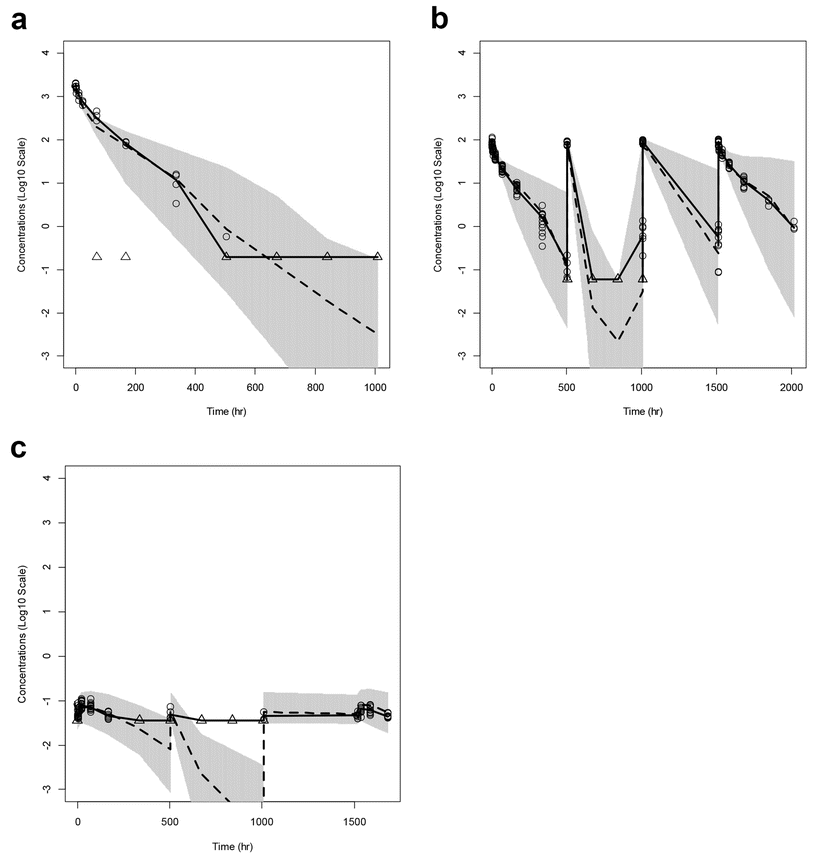

Supplement: Supplementary file 1 — (GIF 39 kb) [file 11095_2014_1585_Fig10_ESM.gif]

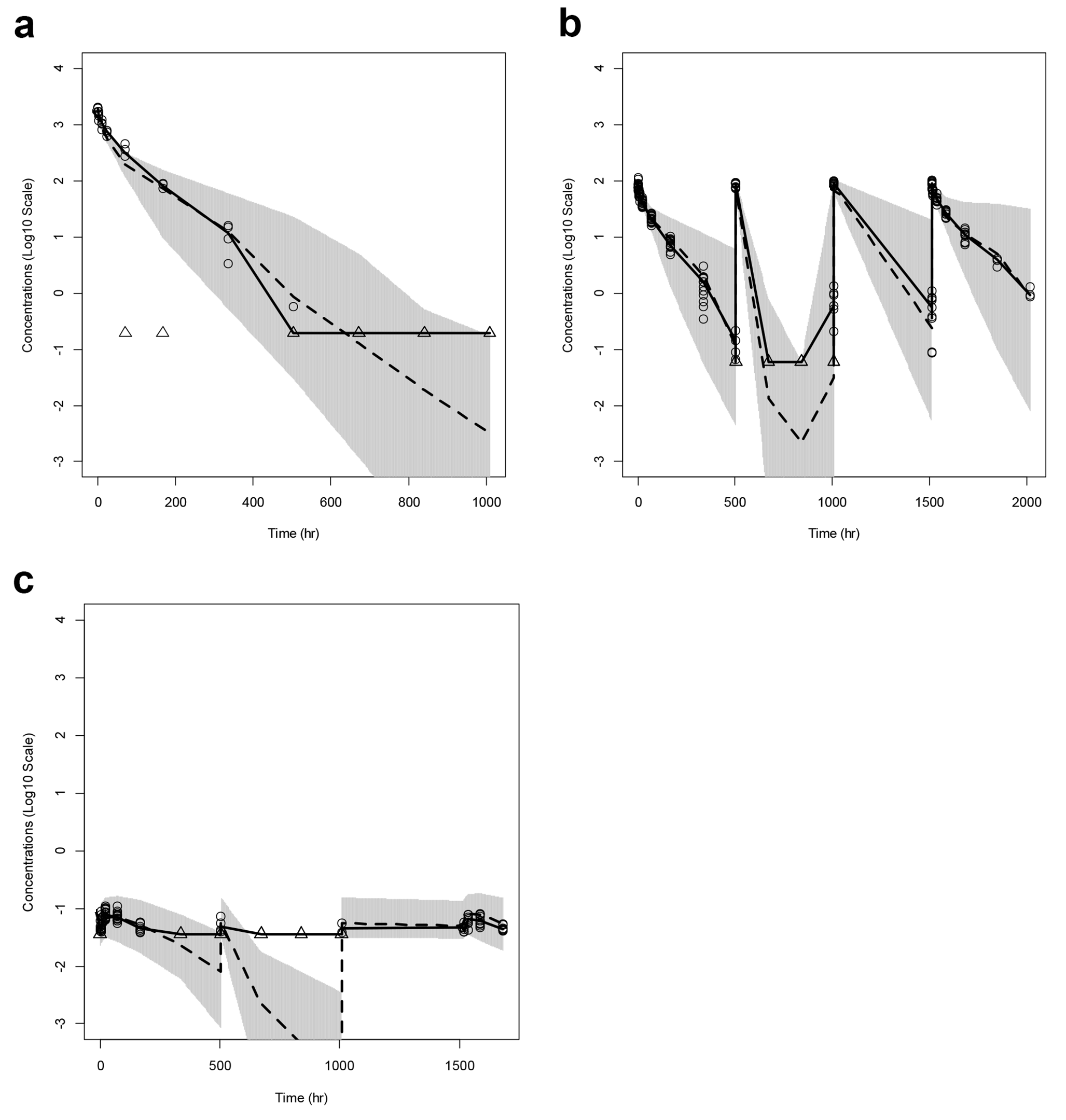

Supplement: Supplementary file 2 — High resolution image (TIFF 707 kb) [file 11095_2014_1585_MOESM1_ESM.tif]
